# Experimental Investigation on Cutting Force and Hole Quality in Milling of Ti-6Al-4V

**DOI:** 10.3390/mi17010019

**Published:** 2025-12-24

**Authors:** Laifa Zhu, Kechuang Zhang, Bin Liu, Feng Jiang, Xian Wu, Lulu Zhai, Fuping Huang, Wenbiao You, Tongtong Xu, Shanqin Zhang, Rongcheng Guo, Yipeng Xue, Xiaoya Chen

**Affiliations:** 1 College of Mechanical Engineering and Automation, Huaqiao University, Xiamen 361021, China; 2 Key Laboratory of Complex Flow and Fluid Engineering Equipment of Zhejiang Province, Zhejiang Sci-Tech University, Hangzhou 310018, China

**Keywords:** milling, Ti-6Al-4V, cutting force, burr formation, hole quality

## Abstract

High-quality hole machining of Ti-6Al-4V is critical for precision aerospace components but remains challenging due to the alloy’s poor machinability. In this study, the influence of cutting parameters on milling force, burr formation and the hole quality of Ti-6Al-4V was investigated. The mechanical properties and microstructure of the milled holes were analyzed. The research results show that milling depth is the primary factor governing variations in milling force and burr formation. The minimum milling force of 3.61 N is achieved at a milling depth of 60 μm, a feed per tooth of 2 μm/z and a cutting speed of 31 m/min. Compared to pre-optimization parameters, the milling force is decreased by 91.74%. Correspondingly, entrance burr width and hole-axis deviation were substantially reduced, indicating marked improvement in hole quality and geometrical accuracy. Microstructural observations show no deleterious phase transformations or excessive work-hardening under the optimized regime. The results deliver quantitative guidelines for parameter selection and tool application in micro-hole milling of Ti-6Al-4V and provide a foundation for further process modelling and optimization for aerospace manufacturing.

## 1. Introduction

The manufacturing of high-precision components in the aerospace and medical industries increasingly relies on advanced machining technologies [1,2,3]. Among these, milling stands out for its ability to create complex three-dimensional features with high dimensional accuracy. Titanium alloy Ti-6Al-4V, renowned for its high specific strength, excellent corrosion resistance and good biocompatibility, is a cornerstone material in these sectors [4,5,6]. However, these same properties, coupled with poor thermal conductivity and a strong chemical affinity for tool materials, render it notoriously difficult to machine, particularly at the micro-scale [7]. This challenge is further amplified when machining high-aspect-ratio micro-features, which are critical in applications like fuel injection nozzles and cooling channels for advanced turbine blades.

Within the realm of hole machining, the sequential process of drilling followed by micro-milling for hole-enlargement presents distinct advantages over traditional drilling followed by reaming, particularly for achieving high-precision, high-efficiency holes in Ti-6Al-4V. Milling offers superior geometric control and flexibility, enabling not only precise diameter control but also the correction of deviations inherent in the initial drilling process, a capability generally absent in the more limited reaming operation. In the process of hole machining, the use of tools with a large length-to-diameter ratio will lead to reduced rigidity and increased susceptibility to tool deflection and chatter vibrations [8,9]. These dynamic phenomena significantly influence fundamental machining outcomes, including cutting forces, which are a primary indicator of process stability and a key driver of tool wear. Considerable research efforts have been dedicated to modeling and understanding cutting forces in milling processes. Wojciechowski [10] developed a novel predictive force model that explicitly accounts for the effects of chip thickness accumulation in milling. Complementary to this, Tan [11] conducted finite element simulations to investigate workpiece deformation and mechanical behavior during the milling of titanium alloys, while Zheng [12] experimentally studied the associated thermomechanical coupling effects under high-speed milling conditions. Furthermore, Moges [13] presented a comprehensive model incorporating critical milling specific phenomena such as tool runout, elastic recovery of the workpiece material and the minimum chip thickness effect on the resulting cutting forces. A comprehensive understanding of how machining parameters influence the micromechanics of chip formation and the resultant forces is therefore crucial for process optimization and the prevention of premature tool failure [14]. While previous studies have established general force trends in macro-milling, their applicability to the micro-scale, where phenomena like the minimum chip thickness effect dominate, remains limited, especially for high-aspect-ratio geometries in difficult-to-cut materials.

Another persistent issue in micromachining, particularly in ductile materials like Ti-6Al-4V, is the formation of burrs at the hole entrance. Burrs represent a significant reduction in part quality, often necessitating costly and time-consuming secondary deburring operations [15,16]. In precision components, their presence can impair functional performance, interfere with assembly and act as potential initiation sites for fatigue cracks. Research efforts have been directed towards understanding and controlling this phenomenon. Aurich [17] systematically analyzed the burr formation process in milling, providing foundational insights into its mechanisms. Building on this, Kumar [18] developed a model to predict burr height in Ti-6Al-4V after high-speed milling, reinforcing that cutting parameters are a decisive factor influencing burr formation and that their optimization is key to burr reduction and prevention. Further elucidating the underlying material behavior, Ni [19] demonstrated that increased burr formation is linked to enhanced material ductility resulting from dominant ploughing action under certain cutting conditions. The size and morphology of these burrs are intricately linked to the material’s plastic flow behavior at the exit edge of the cutting path, which is governed by the local stresses and temperatures determined by the cutting parameters [20]. A systematic investigation into the parameter-dependent mechanisms of burr formation is a necessary step towards its effective suppression.

Beyond cutting forces and burr formation, the final geometrical accuracy of the hole—encompassing dimensional error, circularity error and perpendicularity—is paramount for ensuring component functionality. Existing research has established that cutting parameters significantly influence these geometrical characteristics. Saptaji [21] identified machining parameters as decisive factors affecting dimensional accuracy, micro-damage and surface errors in micromachining. Furthermore, the prevalent issue of vibrations, exacerbated by size effects and complex milling kinematics, has been highlighted as a major source of inaccuracy. Balazs [22] investigated the substantial impact of these self-excited vibrations in milling. To address this, Wang [23] developed a machining quality model for titanium alloy milling that explicitly incorporates the influence of milling vibrations. However, the combination of low system rigidity and the complex interplay of cutting parameters can lead to substantial deviations from the intended geometry in deep milling [24]. Tool deflection under radial forces can cause hole shrinkage and taper, while dynamic instabilities can manifest as poor roundness. Therefore, fundamental research must be conducted to achieve the stringent tolerances required for critical components. This research will establish the relationship between process parameters and the resulting geometric errors, elucidating the underlying mechanisms responsible.

While the aforementioned studies provide valuable insights into force modelling, burr prediction, and vibration effects in milling, the interplay between parameter-induced forces, dynamic stability, and final hole quality in this challenging machining scenario remains inadequately explored. Therefore, the objective of this work is to conduct a designed experimental investigation to elucidate the effects of cutting depth, feed per tooth, and cutting speed on these critical performance metrics, identify dominant factors and optimal ranges, and analyze the underlying microstructural changes to provide mechanistic insights to bridge this gap.

This study presents a systematic experimental study on the milling of holes in Ti-6Al-4V. A structured orthogonal experimental design is employed to quantitatively investigate the effects of cutting depth, feed per tooth and cutting speed. This study first analyzes the influence of these parameters on the three-component cutting forces and identifies an optimized parameter set for force minimization. The correlation between the micro-morphology of the entrance burr and cutting conditions is determined by characterizing its microstructure and quantifying its width. It is important to note that this study focuses on the micro-milling of blind (non-through) micro-holes. Consequently, the analysis of burr formation is confined to the hole entrance, and phenomena specifically associated with tool exit from a through-hole are not considered here. Furthermore, the resulting holes are evaluated based on diameter error, circularity and perpendicularity, linking geometric inaccuracies to underlying process mechanics. The findings from this work are expected to provide a valuable experimental database and mechanistic insights for the selection of optimal milling parameters, ultimately enhancing the manufacturing capability for high-quality titanium alloy components.

## 2. Experimental Setup

### 2.1. Workpiece Material and Cutting Tool

The critical components of aero-engines are primarily manufactured from titanium alloy Ti-6Al-4V. This material is selected for its exceptional properties, including high specific strength, excellent resistance to elevated temperatures and superior corrosion resistance, making it an ideal choice for demanding aerospace applications [25,26]. In this study, Ti-6Al-4V was employed as the workpiece material for the milling experiments. The key physical properties of the Ti-6Al-4V alloy used are summarized in Table 1.

Carbide end mills are widely recognized as high-performance tools for machining titanium alloys due to their high hardness and exceptional wear resistance, which are prerequisites for achieving high machining accuracy and superior hole quality [27,28]. The tool used in this investigation was a cemented carbide end mill supplied by Xiamen Golden Egret Special Alloy Co., Ltd. in Xiamen, China. This tool was manufactured from WC-Co cemented carbide, and its cutting edges were uncoated. It features a cutting diameter of 2.0 mm and a flute length of 6.0 mm. The tool is equipped with four cutting edges and a unique double helix angle structure with two helix angles of 40° and 42°. It can be used for both side milling and face milling. The combination of a small cutting diameter and a relatively long flute length contributes to reduced cutting forces, thereby enhancing process stability and machining precision [29]. Prior to the experiments, the cutting edges were inspected using an optical microscope (VHX1000, Osaka, Japan) to confirm their integrity and the tool nose radius *r_ε_* and cutting-edge radius *r_β_* were measured. The principal geometric parameters of the tool are detailed in Table 2. This specific tool geometry not only ensures significant improvement in workpiece quality but also guarantees consistent and stable cutting performance over prolonged use.

### 2.2. Milling Experimental Procedure

**Figure 1:**
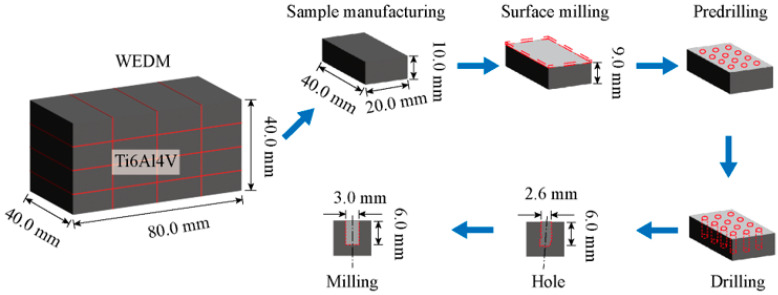
Schematic diagram of milling process for holes with a diameter twice that of titanium alloy Ti-6Al-4V.

The experimental procedure for the milling of holes in Ti-6Al-4V is illustrated in Figure 1. It consists of four main steps: sample manufacturing of the Ti-6Al-4V material, surface milling, drilling and milling for hole enlargement.

**Figure 2:**
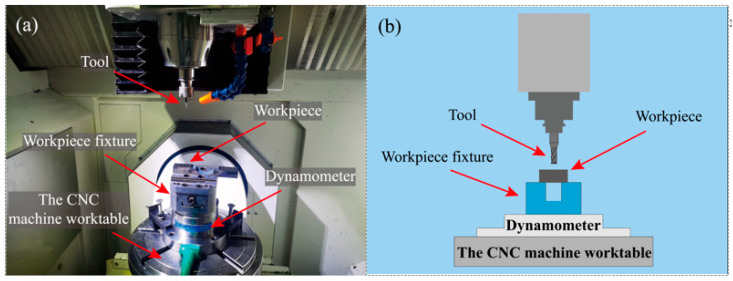
Milling experiment of titanium alloy Ti-6Al-4V. (**a**) Milling experiment. (**b**) Experimental diagram.

Prior to the experiment, the Ti-6Al-4V workpiece was manufactured into samples with dimensions of 40 mm × 20 mm × 10 mm using a wire cutting machine (M735, Suzhou, China) employing Wire Electrical Discharge Machining (WEDM) [30]. To eliminate potential contamination from WEDM residues, the samples were ultrasonically cleaned for 20 min to remove adhering oils and other impurities. After cleaning and drying, the sample was securely mounted onto a piezoelectric dynamometer (9272, Winterthur, Switzerland), which was itself fixed on the CNC machine worktable of a five-axis high-speed machining center (JDGR-200T, Beijing, China). The experimental setup for the Ti-6Al-4V milling is shown in Figure 2. The machining center employed offers a positioning accuracy of 2 μm, a repeatability of 1.8 μm and a maximum spindle speed of 32,000 r/min. Initially, a surface milling operation was performed on the original sample surface using a 10 mm diameter carbide end mill with parameters set to a cutting speed of 57 m/min, a feed per tooth of 2 μm/z and a milling depth of 50 μm. This step ensured surface flatness, removed the oxide layer and guaranteed precise depth control during subsequent milling. Subsequently, a hole was created using a 2.6 mm diameter carbide drill bit at a cutting speed of 16 m/min, a feed rate of 60 mm/min and a pecking depth of 0.6 mm. This process ultimately produced a guide hole measuring 2.6 mm in diameter and 6.0 mm in depth.

The milling experiments were then conducted on the same machining center. A 2 mm diameter carbide end mill was used to mill the pilot hole to a final diameter of 3 mm and a depth of 6 mm and an aspect ratio of two was achieved. The cutting parameters of milling are detailed in Table 3. All milling experiments were repeated three times to ensure statistical reliability.

Milling forces during milling are continuously measured using a piezoelectric multicomponent dynamometer (9272, Kistler, Switzerland). The system features a high natural frequency of 3.5 kHz with a sampling frequency set at 10 kHz. It achieves a minimum resolution of 0.01 N and can accurately capture minute force variations during milling processes. For each experiment, the peak-to-valley values from the stable cutting phase were determined and their average was taken as the final measured cutting force. The milling machining process was conducted under a water-based coolant supply.

**Figure 3:**
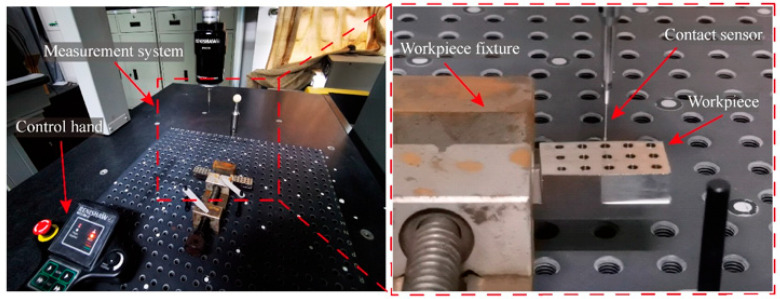
Hole geometric precision inspection system.

To further evaluate the quality of the holes, the burr morphology was examined using a tungsten filament scanning electron microscope (SEM, JSM-IT500, Tokyo, Japan). Furthermore, the geometric accuracy of the holes was measured using a coordinate measuring machine (CMM, GLOBAL-FX777, North Kingston, RI, USA) equipped with a 1.0 mm diameter touch-trigger probe. The system used for hole geometric precision inspection is depicted in Figure 3. Following the CMM measurements, the entrance morphology of selected holes was further observed via SEM to correlate geometric errors with surface characteristics.

### 2.3. Milling Machining Mechanism

**Figure 4:**
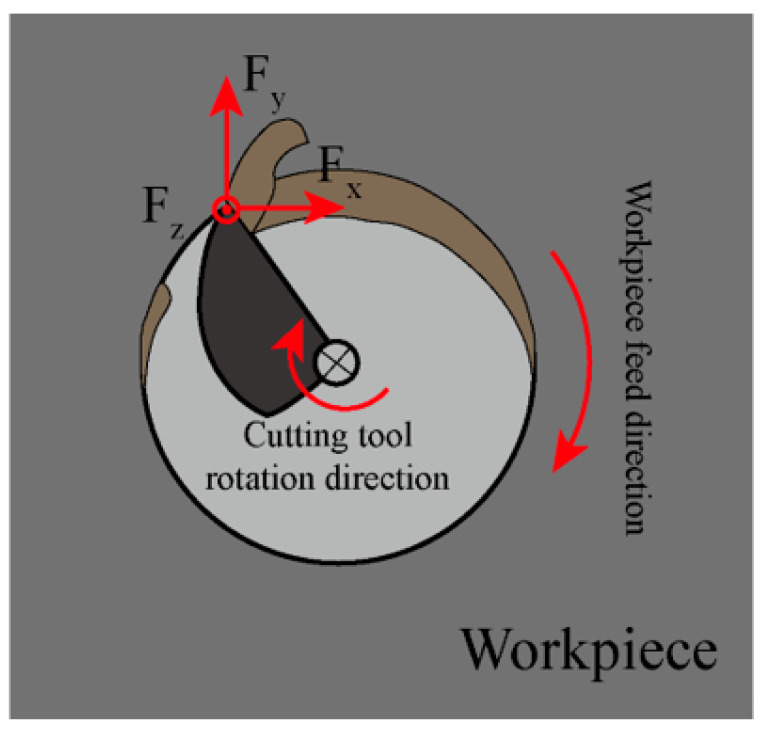
Schematic diagram of the milling force in milling.

Cutting forces serve as a critical indicator of the dynamic characteristics inherent in the machining process. It indirectly reflects the final surface quality and provides vital insights into the cutting state [31]. The force components acting on the workpiece during milling are depicted in Figure 4, where *F_x_* represents the tangential force component, *F_y_* represents the radial force component and *F_z_* represents the axial force component. Due to the periodic engagement and disengagement of the cutting edges with the workpiece, these force components exhibit periodic fluctuations. Based on their characteristics, milling forces can be categorized into static, quasi-dynamic and dynamic components [32]. The static force typically refers to the mean force, while the quasi-dynamic force is characterized by the Peak-to-Valley (P-V) value. The dynamic force is often associated with machining instability. It exhibits randomness and tends to cause abnormal fluctuations in force values.

In milling, the mean force, P-V value and Root Mean Square (RMS) value are commonly used for quantitative force evaluation [33,34]. Due to the intermittent nature of the cutting process, the periodic fluctuation of the cutting force is a fundamental characteristic of milling. This dynamic feature is not adequately captured by either the mean or RMS values [35]. Furthermore, these metrics can be significantly influenced by the thermal drift of the dynamometer and vibrations during milling [36]. Consequently, the quasi-dynamic force represented by the P-V value is considered the optimal parameter for a comprehensive assessment of the milling force. The resultant force F can be calculated using Equation (1), while the P-V force *F_P-V_* is determined by Equation (2). Here, *F_x_* denotes the tangential force, *F_y_* represents the radial force and *F_z_* signifies the axial force. *F_P_* represents the peak force and *F_V_* denotes the valley force.

(1) $$F=\sqrt{{F_{x}}^{2}+{F_{y}}^{2}+{F_{z}}^{2}}$$

(2) $$F_{P-V}=F_{P}-F_{V}$$

**Figure 5:**
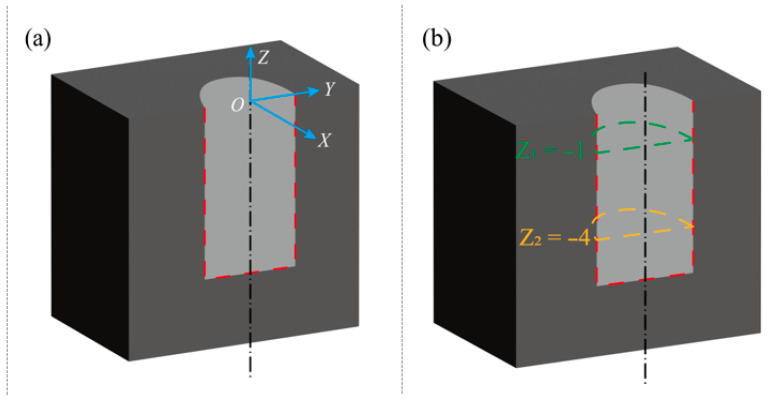
Hole profile diagram. (**a**) Establish coordinate system. (**b**) Measurement planes.

To accurately determine the actual hole diameter, circularity error and perpendicularity of the holes, diameter measurements were conducted at different cross-sectional planes. A coordinate system was established with its origin at the intersection of the hole’s central axis and the workpiece top surface, as illustrated in Figure 5a. The initial probe coordinate was set at (0, 0, 1). Two measurement planes, *Z*_1_ = −1 and *Z*_2_ = −4, were selected for analyzing the geometric accuracy of the hole. A vertical spacing of 3.0 mm was established between the two measurement planes, as shown in Figure 5b. Prior to measurement, the workpiece was securely fixed to ensure its axis remained vertical.

**Figure 6:**
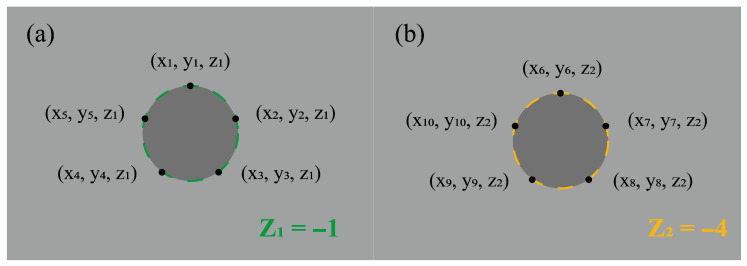
Coordinates of the detection points on the two detection planes (**a**) *Z*_1_ = −1. (**b**) *Z*_2_ = −4.

As the probe moved downwards from (0, 0, 1) along the negative Z-axis, the coordinates of five points distributed around the hole’s circumference at each measurement plane were recorded following the pattern shown in Figure 6. For a given plane *Z*_1_ = −1, the coordinates of the five points are denoted as (*x_i_*, *y_i_*, *z*_1_), where *i* = 1, 2, 3, 4, 5 and (*x*_6_, *y*_6_, *z*_1_) is considered equal to (*x*_1_, *y*_1_, *z*_1_). The chord length *s_i_* between adjacent points can be calculated using Equation (3).

(3) $$s_{i}=\sqrt{{\left(x_{i+1}-x_{i}\right)}^{2}+{\left(y_{i+1}-y_{i}\right)}^{2}}.$$

(4) $$s=2r\sin\frac{\theta }{2},$$

Given the uniform distribution of the points, the central angle *θ* subtended by adjacent chords is 72°. Utilizing the relationship between chord length and radius in Equation (4), the radius *r_i_* for each chord can be derived from Equations (3) and (4).

(5) $$r_{i}=\frac{s_{i}}{2\sin\frac{\theta }{2}},$$

(6) $$d_{i}=2r_{i},$$

(7) $$d_{i}=\frac{s_{i}}{\sin\frac{\theta }{2}},$$

(8) $$\bar{d_{Z1}}=\frac{1}{5}\sum_{i=1}^{5}d_{i}.$$

**Figure 7:**
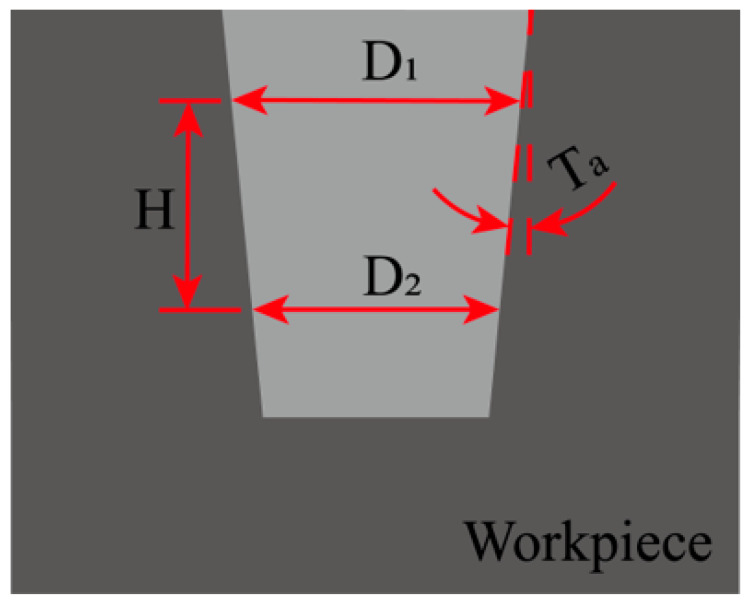
Measurement of circularity error and taper angle.

The corresponding diameter *d_i_* is then calculated via Equations (5)–(7). The average diameter $\bar{d_{Z1}}$ for the plane at *Z*_1_ is obtained by averaging these five calculated diameters *d_i_* as per Equation (8). The same procedure is applied to determine the average diameter $\bar{d_{Z2}}$ at the *Z*_2_ = −4 plane. From these measurements, the maximum hole diameter $D_{1}=max\left\{\bar{d_{Z1}}\right.,\;\left.\bar{d_{Z2}}\right\}$ and the minimum hole diameter $D_{2}=min\left\{\bar{d_{Z1}}\right.,\;\left.\bar{d_{Z2}}\right\}$ for each hole are identified. The roundness error *E_c_* and the perpendicularity *T_a_* of the hole in Ti-6Al-4V are then calculated using Equations (9) and (10) with the measurement schematic provided in Figure 7.

(9) $$E_{c}=\left|\frac{D_{1}+D_{2}}{2}-D_{0}\right|,$$

(10) $$T_{a}=\tan^{-1}\frac{D_{1}-D_{2}}{2H}\times \frac{180}{\pi }.$$

In Equations (9) and (10), *E_c_* is the roundness error, *T_a_* is the taper angle, *D*_0_ is the standard hole diameter of 3.0 mm, *D*_1_ is the maximum measured diameter, *D*_2_ is the minimum measured diameter and *H* is the vertical distance between planes *Z*_1_ and *Z*_2_, which is 3.0 mm. The raw coordinate data from the CMM were processed offline using Equations (3)–(10) to calculate the average hole diameters *d*, circularity error *E_c_*, and taper angle *T_a_*.

## 3. Results and Discussion

### 3.1. Effect of Milling Parameters on Milling Force and Parameter Optimization

During the milling of Ti-6Al-4V, the cutting tool is subjected to a tri-axial force system comprising tangential, radial and axial components. Consequently, both milling performance and the resultant geometrical accuracy of the holes are directly correlated with the magnitude of these cutting forces. Building upon the aforementioned foundation, this section investigates the influence of milling parameters using cemented carbide end mills.

**Figure 8:**
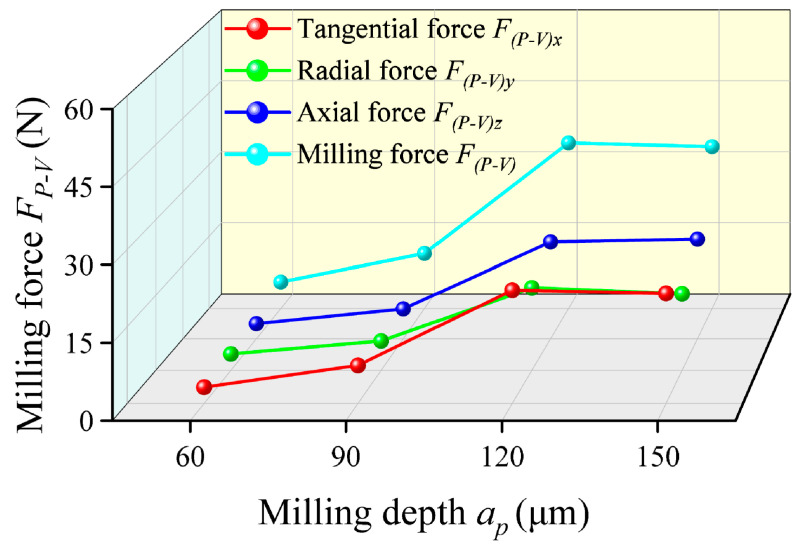
Variation curves of the milling force *F_P-V_* with different milling depths.

The final experimental results, listed in Table 4, encompass various cutting parameters and their corresponding milling forces. The variation in the milling force *F_P-V_* with different milling depths *a_p_* is presented in Figure 8. As the milling depth increases from 60 μm to 150 μm, the tangential force *F_(P-V)x_*, radial force *F_(P-V)y_* and axial force *F_(P-V)z_* components all exhibit a monotonically increasing trend. This is similar to previous research by Zhang [37]. This phenomenon is primarily attributed to the direct increase in material removal rate. A larger milling depth increases the cross-sectional area of the undeformed chip engaged by the cutting edge, thereby elevating the cutting load. This increased load necessitates overcoming greater material resistance during cutting, leading to the observed rise in cutting forces. It is noteworthy that the tangential force *F_(P-V)x_* demonstrates the most pronounced increase. This is attributed to the intensified instantaneous tangential load and potential scale flexible deformation of the slender tool overhang under larger *a_p_* values, which compromises cutting stability. Although a larger milling depth enhances material removal efficiency, the consequent sharp increase in cutting force exacerbates tool wear and induces chatter [38,39,40]. This leads to a decline in hole quality and geometrical accuracy. Within the relatively low-rigidity milling system, selecting a moderate milling depth is paramount for balancing machining efficiency with final quality.

**Figure 9:**
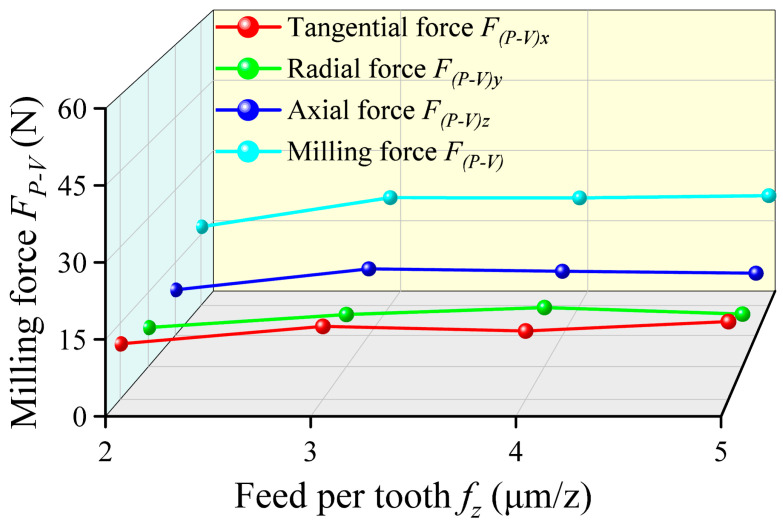
Variation curves of the milling force *F_P-V_* with different feed per tooth.

The feed per tooth *f_z_* significantly influences the material removal mechanism and cutting dynamics in milling, thereby dictating the dynamic characteristics of the milling forces. As shown in Figure 9, the resultant force *F_P-V_* does not exhibit a simple linear relationship as *f_z_* increases from 2 μm/z to 5 μm/z. Instead, it peaks at a feed per tooth of 3 μm/z. This non-monotonic behavior is fundamentally governed by the “minimum chip thickness effect” [41,42]. When *f_z_* approaches or falls below the cutting-edge radius of the mill, the dominant material deformation mechanism transitions from efficient shearing to inefficient ploughing and rubbing [43,44]. At the lower feed of 2 μm/z, the actual uncut chip thickness is insufficient for effective shearing. The cutting edge plastically pushes a significant volume of material sideways and ahead, resulting in abnormally high specific cutting forces [45]. As *f_z_* increases to 3 μm/z, the uncut chip thickness reaches or exceeds the critical threshold for continuous chip formation through shearing. This enables efficient material removal and consequently manifests the peak milling force. With a further increase in *f_z_* to 5 μm/z, the milling force stabilizes or even slightly decreases. This finding was confirmed in Wu’s research [46]. This occurs because stable shear forces become dominant and the plowing effect diminishes, which returns the milling force to its normal level. Furthermore, the increased chip thickness associated with higher feeds can alter strain hardening behavior during chip formation and promote discontinuous chip segmentation, which partially alleviates the continuous accumulation of cutting energy.

**Figure 10:**
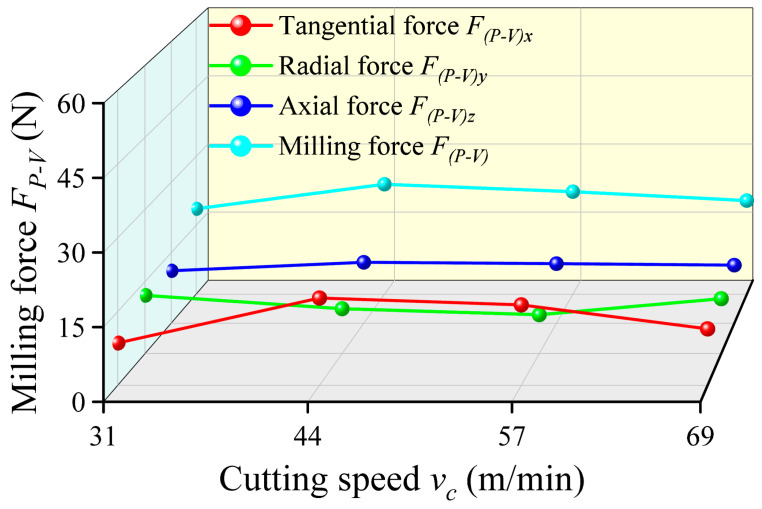
Variation curves of the milling force *F_P-V_* with different cutting speeds.

Cutting speed *v_c_* critically influences the cutting mechanics and consequently affects the dynamic characteristics of the milling process, which are dynamically reflected in the milling forces. As shown in Figure 10, the milling force *F_P-V_* demonstrates a nonlinear trend that first increases and then decreases when the cutting speed rises from 31 m/min to 69 m/min. In the lower speed range of 31 m/min to 44 m/min, the increase in milling force is primarily driven by the dominant effect of strain rate strengthening. As a typical difficult-to-machine material, Ti-6Al-4V is highly sensitive to strain rate. As cutting speed increases, the strain rate in the primary shear zone rises sharply. The flow stress of the material is significantly elevated, which requires greater cutting forces to accomplish shearing.

However, when the cutting speed surpasses 44 m/min, the milling force begins to decline. This phenomenon stems from multiple interrelated factors. Firstly, sufficiently high cutting speeds promote the formation of adiabatic shear bands and discontinuous serrated chips, which effectively reduce the tool-chip contact length and decrease the energy required for plastic deformation. Secondly, despite the low thermal conductivity of titanium alloys, the heat generated in the localized shear zone at high speeds cannot dissipate rapidly. This leads to a significant temperature rise that induces a thermal softening effect in the material. When this thermal softening effect surpasses the strain rate strengthening effect, the overall flow stress of the material decreases, which results in lower milling forces [47,48]. Notably, the tangential force *F_(P-V)x_* exhibits the most significant variation among the three force components. It is important to note that while reduced milling forces and generally improved surface quality are achieved at high cutting speeds, excessively high speeds in milling can exacerbate minor tool runout due to centrifugal forces and accelerate tool wear. This negatively impacts long-term machining stability. Therefore, selecting an optimal cutting speed that balances thermo-mechanical loads and system dynamic stability is crucial for achieving high comprehensive machining quality.

Based on the analysis of milling parameter effects on milling forces, parameter optimization was conducted. The investigation revealed that among the three parameters studied, the milling depth is the predominant factor influencing the variation in milling forces in the milling process, followed by the feed per tooth, while the cutting speed exhibits a relatively minor influence. The maximum resultant force of 43.70 N was observed at the parameter combination of *a_p_* = 150 μm, *f_z_* = 5 μm/z, and *v_c_* = 44 m/min. Analysis of the three-factor four-level orthogonal experimental results identified the optimal parameter combination for minimizing the cutting force as a milling depth of 60 μm, a feed per tooth of 2 μm/z and a cutting speed of 31 m/min. Under these optimized conditions, the resultant milling force reached its minimum value of 3.61 N. Compared to the maximum force condition, the milling force decreased by 91.74%.

### 3.2. Effect of Milling Parameters on Burr Formation

**Figure 11:**
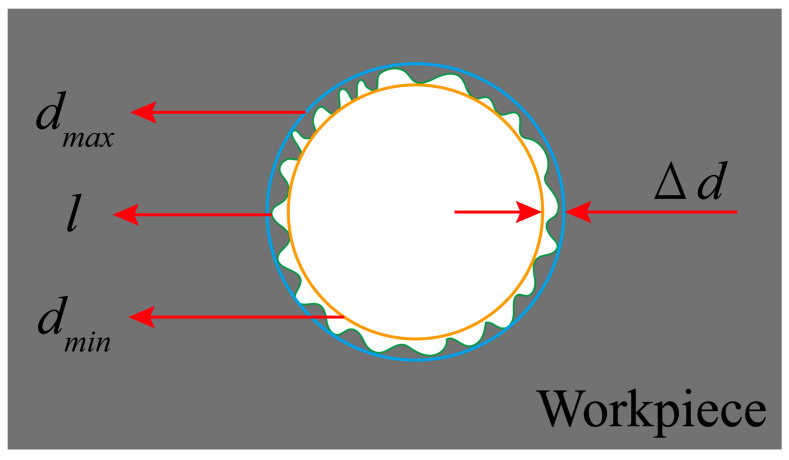
Schematic diagram for measuring burr width.

Figure 11 illustrates the schematic for measuring the burr width Δ*d* at the hole entrance after the milling of Ti-6Al-4V. Here, *d_max_* represents the maximum diameter of the measured burr, *d_min_* represents the minimum diameter of the measured burr and *l* denotes the actual burr profile. The burr width Δ*d* can be calculated using Equation (11).

(11) $$\Delta d=\frac{1}{2}\left(d_{max}-d_{min}\right)$$

**Figure 12:**
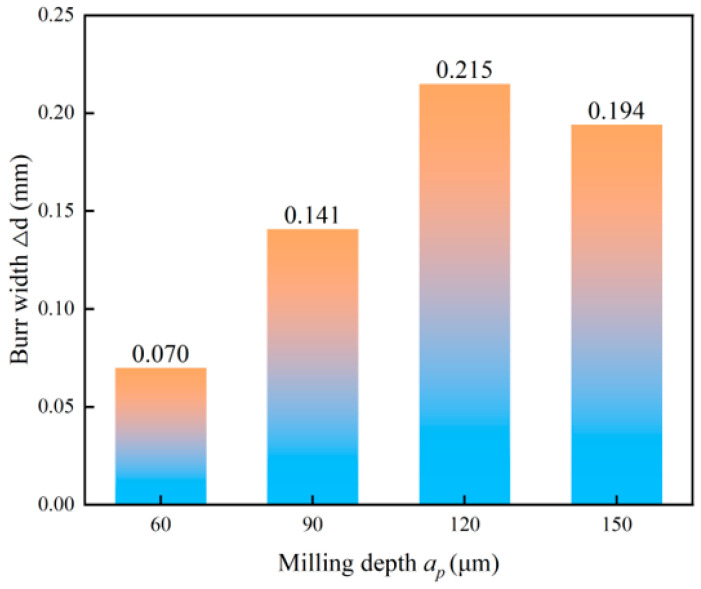
Effect of milling depth on burr width.

**Figure 13:**
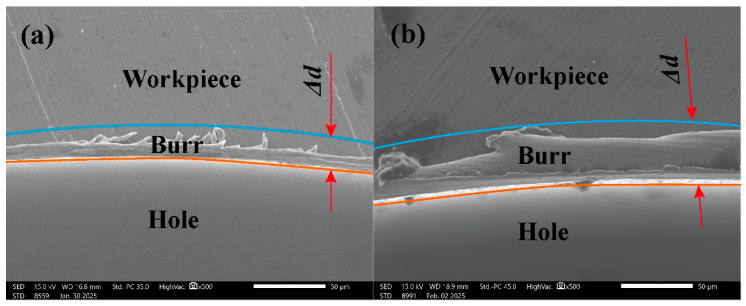

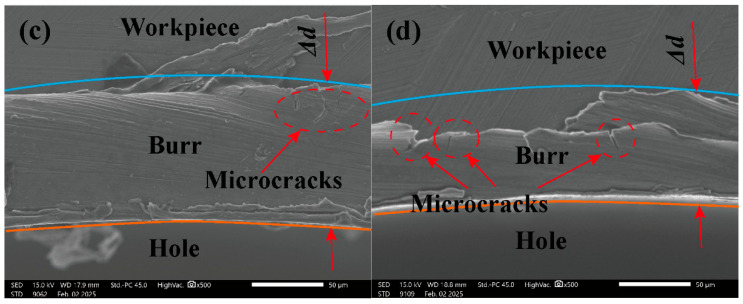
SEM images of burr microstructure under different milling depths. (**a**) *a_p_* = 60 μm. (**b**) *a_p_* = 90 μm. (**c**) *a_p_* = 120 μm. (**d**) *a_p_* = 150 μm.

The variation in burr width Δ*d* with increasing milling depth is shown in Figure 12. The burr width initially increases, reaches a peak at a milling depth of 120 μm and then decreases with a further increase in milling depth. This non-monotonic trend is primarily attributed to the interplay between cutting force and tool/workpiece plastic deformation. The burr microstructure under different milling depths is shown in Figure 13. At moderate milling depths of 60 μm to120 μm, the increased depth of cut elevates the specific cutting force and enhances the plastic flow of the workpiece material towards the exit side of the hole, which leads to more substantial burr formation [49]. Similar descriptions can be found in studies authored by Piquard [50] and Kumar [51]. However, as the milling depth increases from 120 μm to 150 μm, the significantly elevated milling forces and energy input induce greater tool deflection. This shift promotes the transition of the chip formation mechanism towards more extensive plastic deformation or even localized microcracks at the exit edge, as shown in Figure 13c,d. This fracture can potentially break off part of the nascent burr, which results in an apparent reduction in the measured burr width.

**Figure 14:**
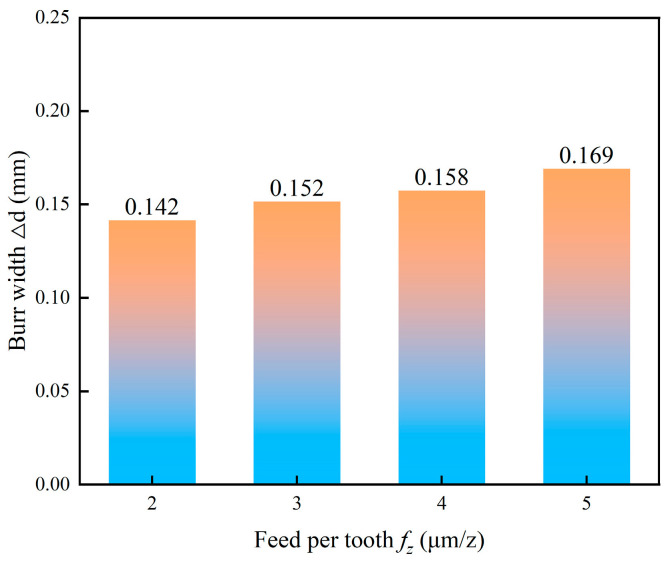
Effect of feed per tooth on burr width.

Figure 14 depicts the relationship between burr width and feed per tooth. It was observed that as the feed per tooth increased, the burr width correspondingly increased with a positive correlation. This relationship is explained by the associated increase in the uncut chip thickness. A larger feed per tooth raises the volume of material being deformed and removed per cutting edge engagement. This amplifies the plastic flow and the side-push effect exerted by the cutting edge on the workpiece material at the hole exit. Consequently, a larger accumulation of plastically deformed material forms at the edge, which manifests as a wider burr.

**Figure 15:**
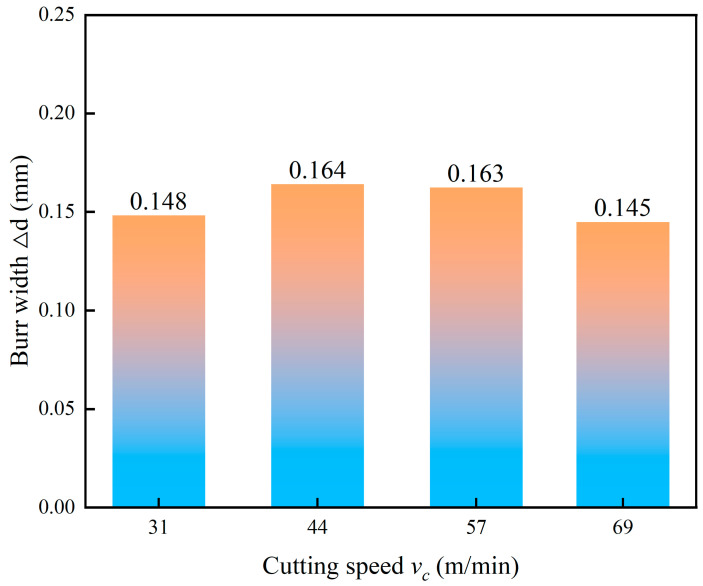
Effect of cutting speed on burr width.

The influence of cutting speed on burr width is shown in Figure 15. The burr width demonstrates a non-linear trend, initially increasing, peaking at 44 m/min and then decreasing with further increases in cutting speed. This behavior is governed by the competing effects of strain rate hardening and thermal softening inherent to Ti-6Al-4V. At lower to medium speeds below 44 m/min, the dominant mechanism is strain rate hardening. The increased deformation rate reduces the material’s ductility and enhances its flow stress, requiring greater cutting forces and promoting more brittle-like fracture behavior at the exit edge, which favors larger burr formation. As the cutting speed increases beyond this point, the combined effects of substantial frictional heat and low thermal conductivity of titanium lead to a concentration of heat within the shear zone. This induces a pronounced thermal softening effect that counteracts the strain rate hardening, reduces the material’s flow stress and improves its localized ductility. This facilitates smoother material separation and shearing at the exit, thereby suppressing plastic side flow and reducing burr dimensions.

### 3.3. Effect of Milling Parameters on Hole Geometrical Accuracy

In milling, the elongated tool overhang required for accessing the hole depth inherently reduces system rigidity, which makes the tool susceptible to minute vibrations. These vibrations often manifest as vibration marks. This can severely deteriorate the geometrical accuracy of machined features, thereby compromising the qualification rate of precision components. To enhance the quality of holes, this section systematically investigates the effects of milling parameters on geometrical accuracy, which covers hole diameter, circularity error and perpendicularity.

**Figure 16:**
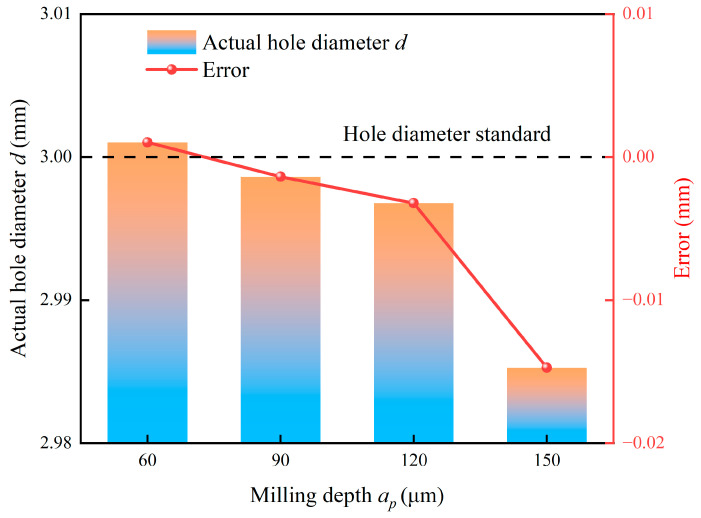
Effect of milling depth on actual hole diameter.

**Figure 17:**
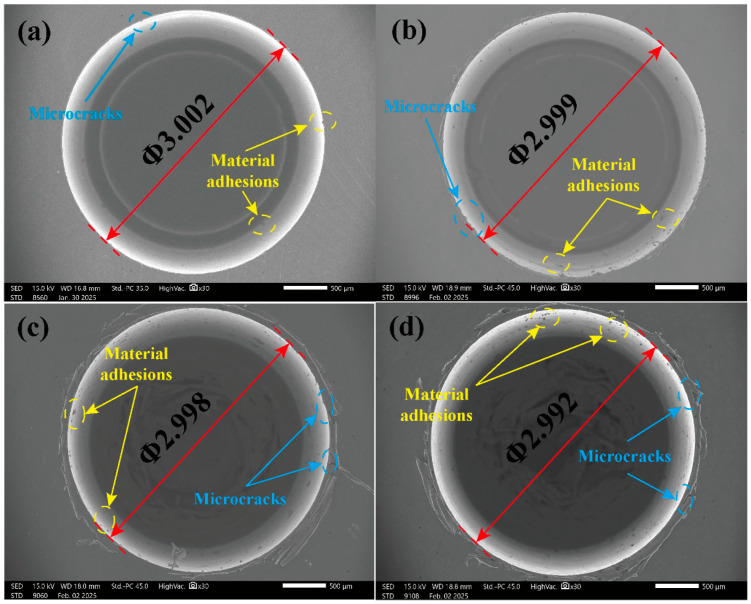
SEM images of actual hole diameter under different milling depths. (**a**) *a_p_* = 60 μm. (**b**) *a_p_* = 90 μm. (**c**) *a_p_* = 120 μm. (**d**) *a_p_* = 150 μm.

Figure 16 compares the actual hole diameter and its deviation under different milling depths. As the milling depth is increased from 60 μm to 150 μm, the actual hole diameter shows a consistent decreasing trend, which leads to a progressive increase in negative deviation from the nominal diameter. This phenomenon is primarily attributable to increased tool deflection. A larger milling depth significantly elevates the radial milling force component. When this force acts on the slender tool with an overhang, it causes significant elastic bending of the tool shank. This deflection displaces the tool axis from its programmed path, effectively reducing the radial engagement and generating an undersized hole. The strong chemical reactivity and high affinity between titanium alloys and cemented carbide cutting tools, coupled with elevated cutting temperatures during micro-milling, facilitate material adhesion and incipient microcrack phenomena at the hole entrance. SEM images of actual hole diameters and microstructures at different milling depths are shown in Figure 17. Furthermore, exacerbated tool wear at greater depths may alter the effective cutting-edge geometry, which contributes to this observed trend.

**Figure 18:**
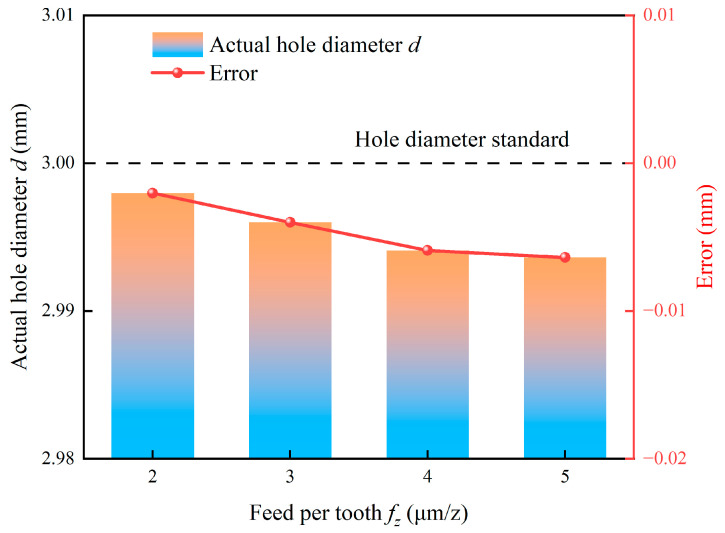
Effect of feed per tooth on actual hole diameter.

The variation in actual hole diameter with feed per tooth is shown in Figure 18. An increase in feed per tooth from 2 μm/z to 5 μm/z similarly leads to a continuous decrease in the actual hole diameter. The fundamental mechanism is similar to that of milling depth and originates from the increase in cutting force. A higher feed per tooth increases the uncut chip thickness, which directly raises the specific cutting force. The consequent increase in the radial force component amplifies tool deflection, thereby magnifying the negative dimensional error. The persistence of this trend across the tested range suggests that the force-induced deflection outweighs any potential mitigating effects from reduced ploughing at higher feeds [52].

**Figure 19:**
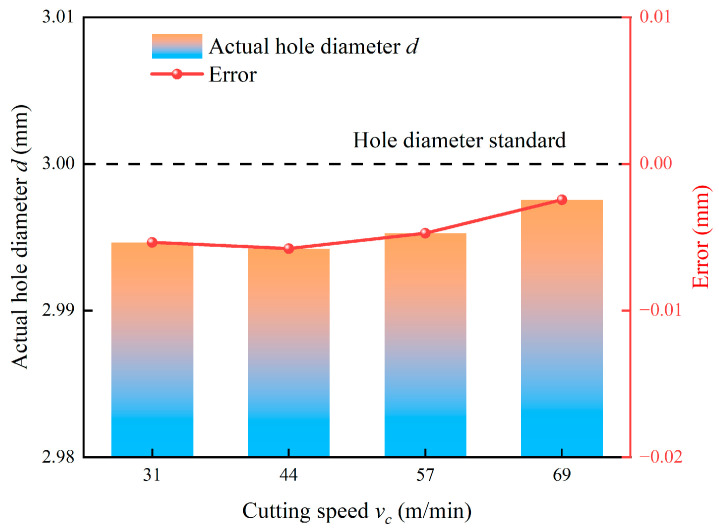
Effect of cutting speed on actual hole diameter.

In contrast, Figure 19 demonstrates that increasing the cutting speed from 31 m/min to 69 m/min results in an increase in the actual hole diameter, effectively reducing the negative error. This improvement is governed by two interrelated factors. First, the dominant thermal softening effect at elevated cutting speeds reduces the material’s flow stress, consequently lowering the overall cutting forces and mitigating tool deflection. Second, higher cutting speeds can shift the cutting process away from the dominant modes of the system’s dynamic response, reducing forced vibrations and promoting a more stable cutting process. This enhanced stability allows the tool to follow its intended trajectory more faithfully.

**Figure 20:**
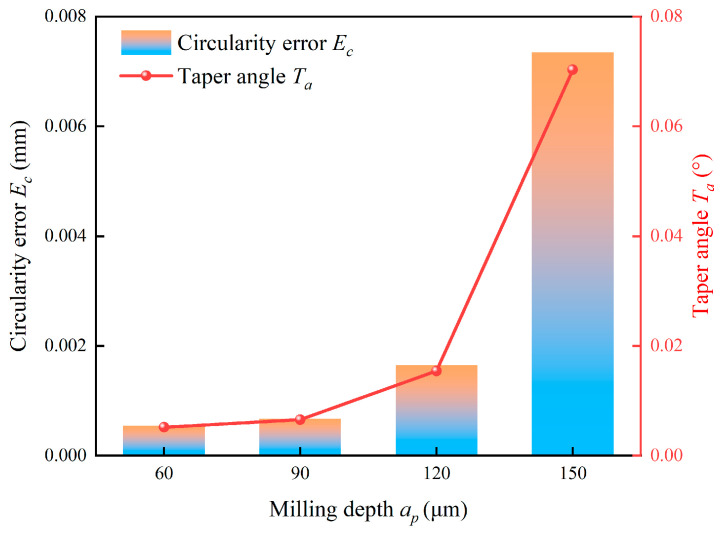
Effect of milling depth on hole quality.

Figure 20 compares hole quality at different milling depths, focusing particularly on circularity error and taper angle. Both error metrics exhibit a continuous increasing trend with rising milling depth and a particularly sharp deterioration is observed between 120 μm and 150 μm. The general trend is a direct consequence of the progressively increasing tool deflection and decreased process stability with depth, which distorts the ideal circular tool path. The abrupt degradation beyond 120 μm likely indicates the onset of significant dynamic instability and chatter. At this critical depth, the combination of high cutting forces and the tool’s natural frequency may trigger intense vibrations, severely compromising hole form accuracy.

**Figure 21:**
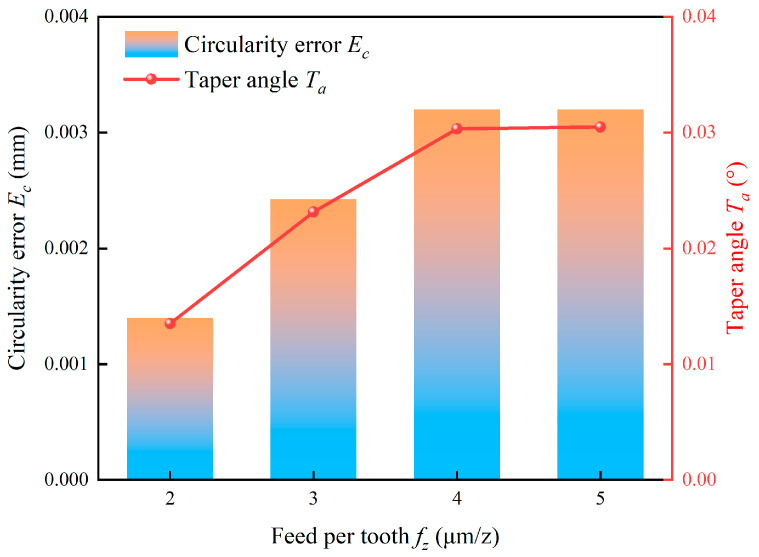
Effect of feed per tooth on hole quality.

The influence of feed per tooth on circularity error and taper angle is shown in Figure 21. Both errors increase linearly with the feed per tooth until they stabilize after 4 μm/z. The initial linear increase is directly correlated with the rising cutting forces, which exacerbate tool deflection and vibration. The subsequent saturation observed at higher feed per tooth indicates that the cutting forces have reached a stable maximum value under the given tool geometry and workpiece material conditions. This result was confirmed by Alemu [53].

**Figure 22:**
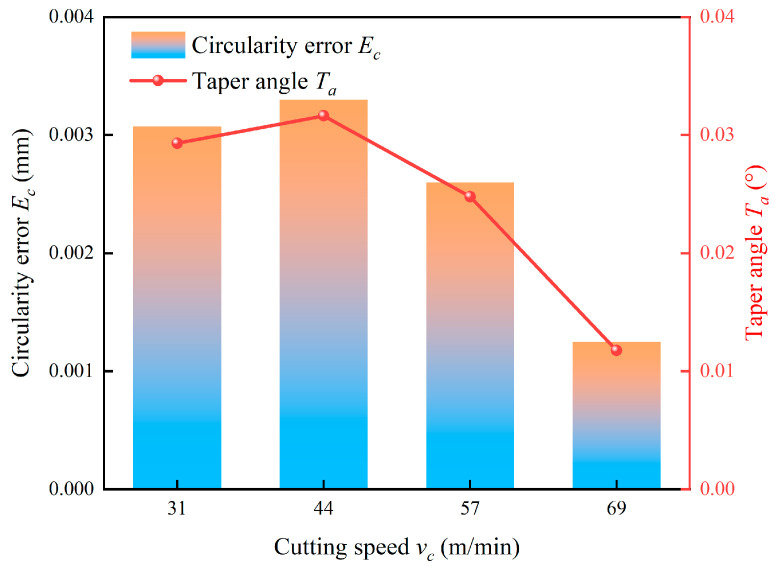
Effect of cutting speed on hole quality.

Finally, Figure 22 illustrates the effect of cutting speed on circularity error and taper angle. The errors demonstrate a non-monotonic trend, initially increasing, peaking at 44 m/min and then decreasing with a further speed increase. This behavior is characteristic of the stability lobe effect in milling processes. The peak errors at 44 m/min likely correspond to a cutting speed that excites a dominant natural frequency of the machining system, which leads to pronounced chatter and poor geometrical integrity. It is particularly noteworthy that the errors decrease sharply as the speed increases from 44 m/min to 69 m/min. This significant improvement occurs because these higher speeds fall outside the resonant frequency band, effectively suppressing chatter vibrations. The concomitant reduction in cutting force due to thermal softening further stabilizes the process, resulting in superior circularity and perpendicularity.

## 4. Conclusions

This study presents a comprehensive experimental investigation into the milling of holes in Ti-6Al-4V alloy, elucidating the effects of milling parameters on milling force, burr formation and geometrical accuracy. The key findings of the study are concluded as follows:

1. Milling force is predominantly governed by the milling depth, which exhibits the most significant influence due to its direct proportionality to the uncut chip cross-sectional area and the resultant tool deflection. Feed per tooth is the secondary factor, while cutting speed demonstrates a non-monotonic influence, where a transition from strain-rate hardening to thermal softening dominance leads to a force reduction at elevated speeds. The optimal parameter combination for minimizing the resultant force to 3.61 N was identified as a milling depth of 60 μm, a feed per tooth of 2 μm/z and a cutting speed of 31 m/min.
2. Burr formation at the hole entrance is critically dependent on the interplay between material plastic flow and the cutting mechanism. Burr width increases with feed per tooth due to the increased volume of plastically flowing material. Conversely, both milling depth and cutting speed exhibit nonlinear relationships with burr size, governed by competing mechanisms. Increasing depth intensifies plastic flow, yet excessive depth may induce fracture. While higher speeds initially promote strain rate hardening, they ultimately suppress burring formation through thermal softening effects and improved material shearing.
3. The geometrical accuracy of holes is primarily determined by the dynamic stability of the machining system, which is heavily influenced by tool deflection and chatter. Larger milling depth and feed per tooth exacerbate tool deflection, leading to undersized holes and degraded form accuracy. The relationship between cutting speed and geometrical errors is characterized by a distinct stability lobe effect. The critical speed of 44 m/min excites system resonance, which causes peak errors. However, higher rotational speeds between 57 m/min and 69 m/min stabilize the machining process and significantly enhance hole quality.
4. Based on the multi-objective analysis, an optimal parameter window for balancing low cutting force, minimal burr formation, and high geometrical accuracy in milling of Ti-6Al-4V is identified: a milling depth of 60–90 μm, a feed per tooth of 2–3 μm/z, and a cutting speed of 57–69 m/min.
5. The research underscores that successful process optimization for high-quality holes requires a multi-objective approach that balances the often-conflicting trends in cutting force, burr suppression and geometrical integrity. The findings provide a crucial experimental database and mechanistic understanding for selecting parameters that ensure precision and reliability in the manufacturing of high-value aerospace components.

## Figures and Tables

**Table 1:** The Ti-6Al-4V material properties.

Property	Value
Density (g/cm^3^)	4.51
Elastic modulus (GPa)	110
Poisson ratio	0.34
Thermal conductivity (W/mK)	7.95
Tensile strength (GPa)	1.01
Yield strength (GPa)	0.88

**Table 2:** Tool structure parameters of milling tools.

Parameter	Value
Diameter *D* (mm)	2.0
Cutting edge length *L_c_* (mm)	6.0
Rake angle (°)	0
Flank angle (°)	7
Bottom inclination angle (°)	15
Tool tip corner radius r_ε_ (μm)	20
Tool cutting edge radius r_β_ (μm)	4

**Table 3:** Cutting parameters of milling.

Parameter	Value
Milling depth *a_p_* (μm)	60, 90, 120, 150
Feed per tooth *f_z_* (μm/z)	2, 3, 4, 5
Cutting speed *v_c_* (m/min)	31, 44, 57, 69

**Table 4:** Experimental result: cutting parameters and milling forces.

No.	Cutting Parameters			Milling Forces			
	Milling Depth *a_p_* (μm)	Feed Per Tooth *f_z_* (μm/z)	Cutting Speed *v_c_* (m/min)	Tangential Force *F_(P-V)x_* (N)	Radial Force *F_(P-V)y_* (N)	Axial Force *F_(P-V)z_* (N)	Milling Force *F_(P-V)_* (N)
1	60	2	31	2.10	2.34	1.77	3.61
2	60	3	44	3.21	2.51	2.82	4.96
3	60	4	57	3.73	3.69	3.76	6.45
4	60	5	69	3.68	4.58	4.02	7.12
5	90	2	44	5.93	4.70	4.41	8.76
6	90	3	31	5.01	6.59	5.53	9.96
7	90	4	69	5.95	6.14	6.85	10.95
8	90	5	57	12.83	6.08	7.64	16.13
9	120	2	57	19.17	8.77	13.45	25.00
10	120	3	69	19.70	19.20	20.67	34.41
11	120	4	31	15.96	22.89	21.23	35.07
12	120	5	44	33.57	15.11	23.56	43.70
13	150	2	69	16.62	15.77	17.95	29.11
14	150	3	57	29.71	13.78	25.91	41.76
15	150	4	44	28.32	15.01	21.12	38.39
16	150	5	31	11.34	16.65	16.15	25.82

## Data Availability

The original contributions presented in this study are included in the article. Further inquiries can be directed to the corresponding authors.
